# Billions of basepairs of recently expanded, repetitive sequences are eliminated from the somatic genome during copepod development

**DOI:** 10.1186/1471-2164-15-186

**Published:** 2014-03-11

**Authors:** Cheng Sun, Grace Wyngaard, D Brian Walton, Holly A Wichman, Rachel Lockridge Mueller

**Affiliations:** 1 Department of Biology, Colorado State University, Fort Collins, CO 80523-1878, USA; 2 Department of Biology, James Madison University, Harrisonburg, VA 22807, USA; 3 Department of Mathematics and Statistics, James Madison University, Harrisonburg, VA 22807, USA; 4 Department of Biological Sciences, The Institute for Bioinformatics and Evolutionary Studies, University of Idaho, Moscow, ID 83844–3051, USA

**Keywords:** Chromatin diminution, Genome size, Transposable elements, Germline-soma differentiation, Copepod

## Abstract

**Background:**

Chromatin diminution is the programmed deletion of DNA from presomatic cell or nuclear lineages during development, producing single organisms that contain two different nuclear genomes. Phylogenetically diverse taxa undergo chromatin diminution — some ciliates, nematodes, copepods, and vertebrates. In cyclopoid copepods, chromatin diminution occurs in taxa with massively expanded germline genomes; depending on species, germline genome sizes range from 15 – 75 Gb, 12–74 Gb of which are lost from pre-somatic cell lineages at germline – soma differentiation. This is more than an order of magnitude more sequence than is lost from other taxa. To date, the sequences excised from copepods have not been analyzed using large-scale genomic datasets, and the processes underlying germline genomic gigantism in this clade, as well as the functional significance of chromatin diminution, have remained unknown.

**Results:**

Here, we used high-throughput genomic sequencing and qPCR to characterize the germline and somatic genomes of *Mesocyclops edax*, a freshwater cyclopoid copepod with a germline genome of ~15 Gb and a somatic genome of ~3 Gb. We show that most of the excised DNA consists of repetitive sequences that are either 1) verifiable transposable elements (TEs), or 2) non-simple repeats of likely TE origin. Repeat elements in both genomes are skewed towards younger (i.e. less divergent) elements. Excised DNA is a non-random sample of the germline repeat element landscape; younger elements, and high frequency DNA transposons and LINEs, are disproportionately eliminated from the somatic genome.

**Conclusions:**

Our results suggest that germline genome expansion in *M. edax* reflects explosive repeat element proliferation, and that billions of base pairs of such repeats are deleted from the somatic genome every generation. Thus, we hypothesize that chromatin diminution is a mechanism that controls repeat element load, and that this load can evolve to be divergent between tissue types within single organisms.

## Background

Chromatin diminution is the programmed deletion of DNA from presomatic cell or nuclear lineages during development, producing single organisms that contain two dramatically different nuclear genomes. Phylogenetically diverse taxa undergo some form of chromatin diminution or chromosome elimination, including representatives from the ciliates, nematodes, copepods, lampreys, and hagfish [1-7]. Cyclopoid copepods excise more than an order of magnitude more sequence than other taxa; depending on species, ~12 to 74 Gb of DNA are lost from the presomatic cell lineage at an early embryonic cleavage division each generation [8,9]. This excision produces somatic genomes that are only ~1% – 20% of the size of the germline genomes. Post-diminuted somatic genomes are comparable in size to genomes of related copepods that lack chromatin diminution, whereas the pre-diminuted germline genomes are 5- to 75-fold larger [8,9].

Different functions for chromatin diminution have been proposed for different taxa, reflecting specific properties of the excised DNA (when known). For example, reduction in gene misexpression in the soma through excision of germline-specific genes has been proposed for the lamprey and the nematode *Ascaris*[5,10,11]. In contrast, transposon elimination from the soma, which enables high-level somatic gene expression based on somatic polyploidy without high levels of TE transcription, has been proposed for ciliates [7,12,13]. In copepods, maintenance of high rRNA gene copy number during embryogenesis has been proposed [14,15], as has removal of short, non-functional sequences [16,17]. Because comparatively little is known about the sequence of excised DNA in copepods, however, most copepod-specific hypotheses for the functional significance of chromatin diminution focus on the effects of higher DNA content in the germline, irrespective of sequence. Such hypotheses include modulation of egg size, body size, developmental rate, and cell division rate, all of which are correlated with genome size in copepods [18-21]. To date, the DNA excised from copepods has not been analyzed using large-scale genomic data [16,17], hindering in-depth study of the processes underlying germline genomic gigantism in this clade, as well as the functional significance of chromatin diminution.

Here, we used high-throughput genomic shotgun data and qPCR to characterize the germline and somatic genomes of *Mesocyclops edax*, a freshwater cyclopoid copepod species with a diploid germline genome of ~15 Gb and a diploid somatic genome of ~3 Gb [22]. Chromosomal fragmentation and excision of DNA occurs at the 5th cleavage division; 12 Gb of sequence is lost from each of the 15 presumptive somatic cells during anaphase. Eliminated DNA appears to be heterochromatic and located primarily in the distal half of chromosomes; chromosome number is constant before and after diminution (n = 14) [22]. Because genome expansion (in the absence of polyploidy) typically reflects the accumulation of transposable elements in eukaryotes, we targeted our study towards identifying and classifying repetitive DNA in the germline and somatic genomes. We show that the majority of both *M. edax* genomes consists of repetitive sequences that are either 1) verifiable transposable elements (TEs), or 2) non-simple repeat elements of likely TE origin. These identified repeats explain more than 90% of the difference in size between germline and somatic genomes. Excised repeats are a non-random sample of the total germline genomic repeat landscape; younger repeats are disproportionately excised from the somatic genome, whereas older repeats are disproportionately retained. Similarly, high-frequency DNA transposon and LINE superfamilies are disproportionately excised from the somatic genome, whereas high-frequency LTR retrotransposons are disproportionately retained. Sequence divergences of repeat elements in both genomes show a skew towards younger elements, indicating recent/ongoing repeat proliferation. Taken together, our results suggest that germline genome expansion in *M. edax* reflects explosive proliferation of repeat elements (including known TEs), and that billions of base pairs of such repeats are deleted from the somatic genome every generation. Thus, we hypothesize that chromatin diminution is a mechanism that controls repeat element load, and that this load can evolve to be highly divergent between tissue types within single organisms.

## Results

### Repeat content of germline and somatic genomes

**Figure 1:**
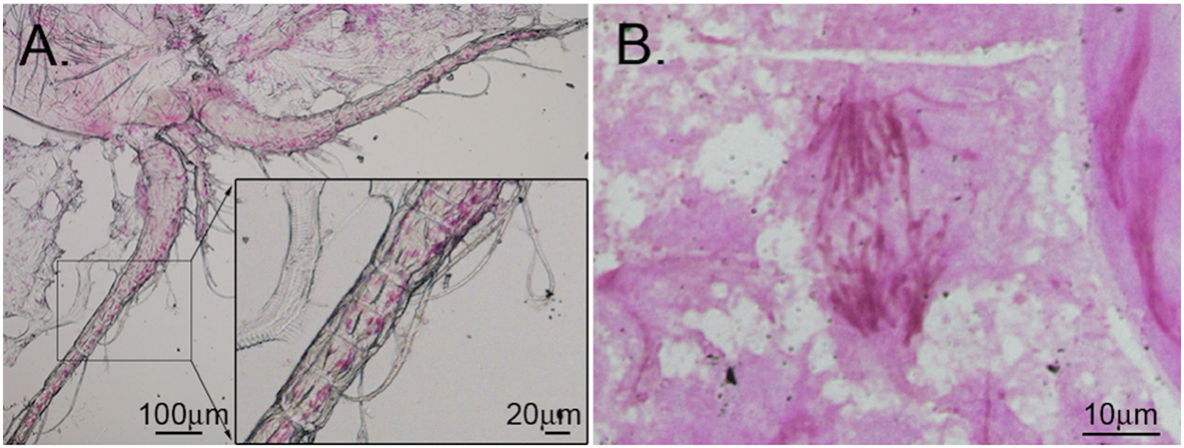
**Feulgen**-**stained nuclei of *M. edax *after and before chromatin diminution. A**. Somatic cell nuclei in antennal segments contain ~3 Gb DNA. **B**. Whole anaphase figure during germline cell division. Germline genome in prediminuted embryo contains ~30 Gb DNA.

We sequenced 0.76% and 1.6% of the germline and somatic genomes, respectively, using amplified genomic DNA from undiminuted embryos and antennae. Summary statistics for shotgun sequencing of genomes from somatic and germline tissue (Figure 1) are summarized in Table 1. All sequences are deposited in the NCBI Sequence Read Archive (Accession numbers: SRR767744 and SRR767746). We modified a pipeline used in our previous studies [23] to mine and classify repeats from low-coverage genomic shotgun data in taxa that lack genomic resources. In total, 87.6% and 71.7% of the total base pairs of shotgun data were masked by the *M. edax*-specific repeat library we generated (see Methods) in the germline and somatic datasets, respectively. Germline and somatic genome sizes are 15 and 3 Gb, respectively. Assuming that these shotgun reads are representative subsets of the whole genomes, ~13.14 Gb of germline DNA and ~2.15 Gb of somatic DNA are composed of the identified repeats, accounting for ~11 of the 12 Gb of DNA eliminated from the presomatic cell lineage during *M. edax* development. Because our methods miss some low-copy-number and highly divergent (i.e. old) repeats, these numbers are underestimates of the true repeat content.

**Table 1:** **Summary statistics from shotgun 454 sequencing of *Mesocyclops edax *** **genomes**

**Genome**	**Genome size**	**reads**	**Average read length**	**% coverage**	**Total basepairs**
Germline	15 Gb	612,470	183	0.76%	112,985,236
Somatic	3 Gb	207,451	216	1.6%	44,863,930

**Figure 2:**
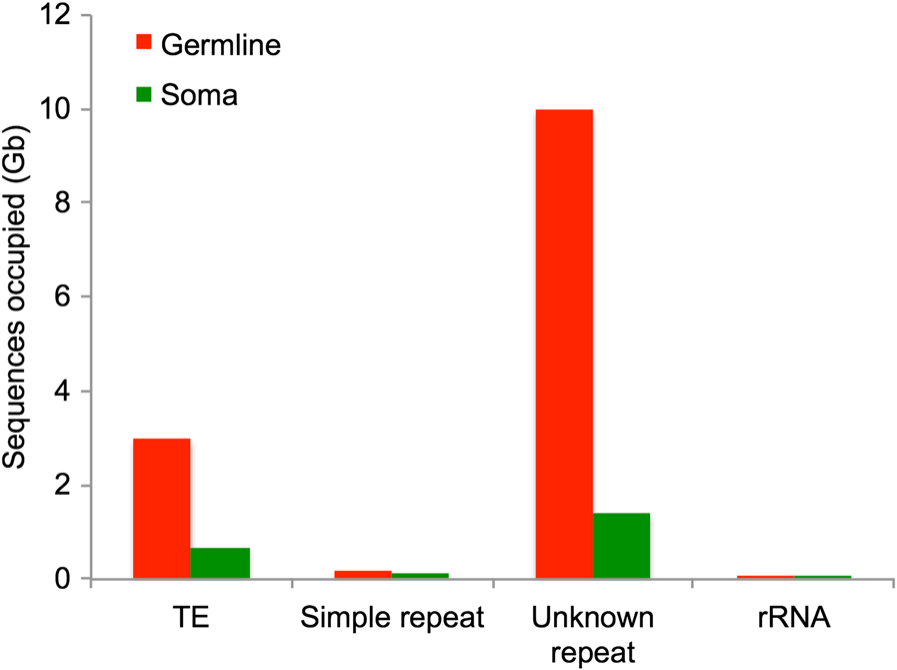
**Germline and somatic genome content.** Estimated Gb of sequence in the germline and somatic genomes annotated as known TEs; simple repeats; unknown, non-simple, non-tandem (within the length of a single read) repeats; and rRNAs. rRNA sequences were only a tiny fraction of both genomes (germline = 0.12%, soma = 0.02%).

Repeats were classified as 1) TEs belonging to known superfamilies (“known TEs”, collectively), 2) simple repeats, 3) unknown repeats, and 4) rRNAs. Figure 2 summarizes the estimated number of base pairs occupied by the different repeat classes in the germline and somatic genomes. TEs and unknown repeats account for the vast majority of the genome size difference between germline and soma; TEs account for ~2.4 Gb and unknowns account for ~8.5 Gb. Simple (including satellite) repeats and rRNA sequences also contribute more sequence to the germline than somatic genomes, but these repeats comprise very small proportions of both genomes.

### Verification of repeat copy number differences between germline and soma with qPCR

**Figure 3:**
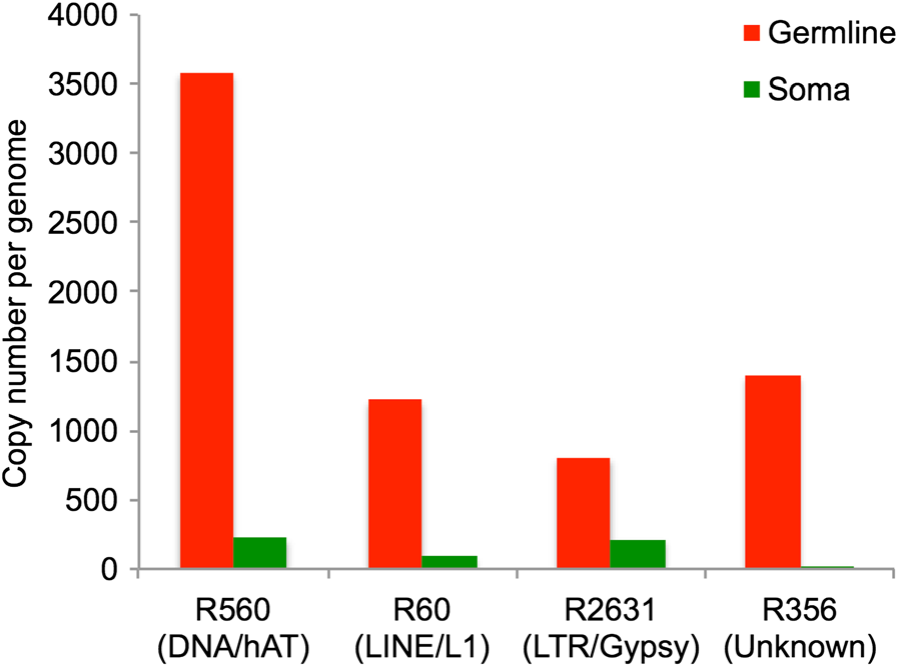
**Copy numbers of four repeat elements in germline and somatic genomes estimated using qPCR.** In all cases, elements are more abundant in the germline than in the soma.

For each of four types of repeats, we picked one representative family that our bioinformatic analyses identified as having higher copy number in germline than soma — DNA/hAT (DNA transposon), LINE/L1 (non-LTR retrotransposon), LTR/Gypsy (LTR retrotransposon), and an unknown repeat. We estimated the copy number of each repeat family in both genomes with quantitative PCR. Results from qPCR analyses are summarized in Figure 3. Germline copy numbers for these four repeats range from 800 – 3,500. Consistent with the bioinformatic results, copy numbers in all cases are higher in the germline than in the soma (3.8 – 62 times higher), indicating that these repeats do contribute to the size difference between the two genomes. We emphasize that our qPCR analyses target individual repeat families; multiple families within each TE superfamily coexist in the genome, each with its own copy numbers in both germline and soma.

### Characterization of unknown repeat sequences

Because unknown repeats make up a substantial portion of both genomes, we performed 1) Open reading frame (ORF) identification, and 2) homology searches to further characterize such sequences. Of the 28,285 unknown repeats present in the *M. edax* repeat library, 4,900 (17.3%) and 2,844 (10.0%) were found to contain ORFs > 100 nucleotides in length using relaxed and strict criteria, respectively. Although most such ORFs were between 100 and 200 base pairs in length, a few were > 600 bp long (Additional file 1). Thus, many of the unknown repeats have the potential to encode proteins. However, only 351 of the 28,285 unknown repeat sequences (1.2%) retrieved hits from the protein database of *Daphnia pulex*, the only crustacean with a sequenced genome to date. Such hits comprised 137 *Daphnia* genes in total and included both hypothetical and known genes (Additional file 2). We note that, because *Daphnia* and *Mesocyclops* last shared a common ancestor hundreds of millions of years ago, this analysis would only detect genes exhibiting long-term sequence conservation, likely reflecting high levels of functional constraint. Thus, although many unknown repeats may encode proteins, most do not appear to be multi-copy endogenous genes exhibiting functional conservation across long evolutionary timescales. Taken together, the presence of ORFs and the lack of recognizable endogenous genes, as well as the non-simple/non-tandem (within the length of a single read) sequence composition, are consistent with the unknown repeats being of TE origin, although more extensive sequencing efforts will be required to classify these repeats conclusively.

### Repeat element sequence divergence in somatic and germline genomes

**Figure 4:**
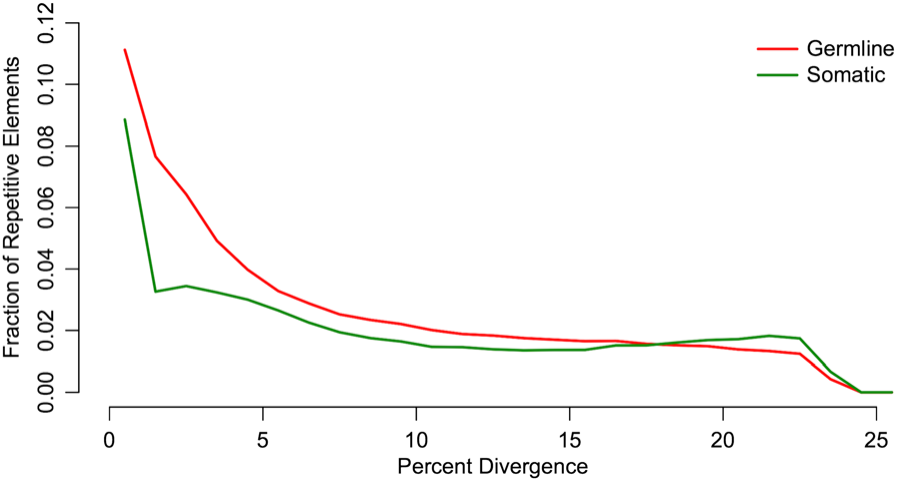
**Sequence divergence distributions of repeats in the germline (red) and soma (green) of *Mesocyclops edax*.** The y axis is the proportion of reads out of the total germline or somatic repeat dataset comprised of repeats of a given sequence divergence/age class, allowing comparisons of the distribution shapes between the two genomes. Sequence divergence distributions of both genomes are skewed towards younger (i.e. less divergent) elements. High proportions of repeats <1% diverged from the consensus demonstrate recent/ongoing repeat proliferation.

To summarize global historical patterns of repeat proliferation and deletion in *M. edax*, we estimated divergences of repeats from their ancestral sequences (i.e. pairwise divergences) in the somatic and germline genomes. Pairwise divergences of repeat elements in both genomes are skewed towards younger (i.e. less divergent) elements, with the highest proportion of repeats showing <1 % divergence from the consensus (Figure 4). Overall, the germline repeats are younger than the somatic repeats. To verify that overall repeat element divergence patterns can be estimated accurately from low-coverage genomic shotgun data, we subsampled the human genome to produce five datasets comparable to our copepod data and estimated pairwise divergence of repeats as described. Repeat pairwise divergence patterns are similar across the five subsampled datasets, showing a proliferation peak in the past and low levels of younger elements (Additional file 3). This result is consistent with the pattern reported from the full human genome sequence [24], confirming that the repeat divergence profile we estimated from *M. edax* with low-coverage shotgun data (particularly the left-skewed pattern) is not an artifact of sequencing coverage. Thus, our results indicate extensive recent and/or ongoing repeat proliferation in *M. edax*[25].

### Characterization of sequences excised during chromatin diminution

**Figure 5:**
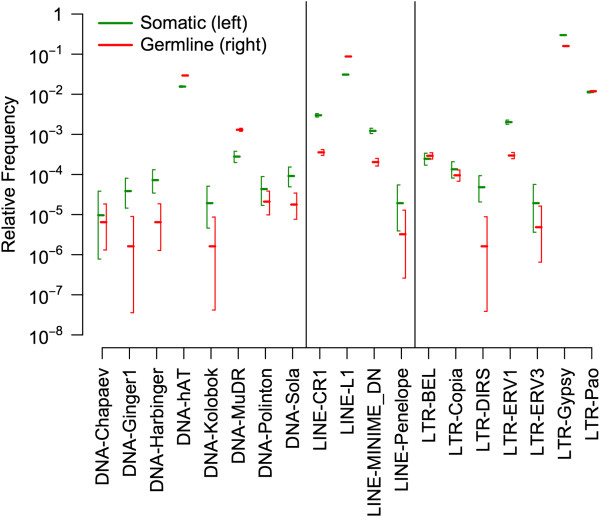
**Maximum likelihood estimates of the relative frequencies of known repeat superfamilies in soma and germline.** 95% confidence intervals are shown. Elements that exist at higher frequencies in the germline than in the soma (e.g. DNA-hAT, DNA-MuDR, LINE-L1) are disproportionately excised from the somatic genome during chromatin diminution. Elements that exist at higher frequency in the soma (e.g. LINE-CR1, LTR-ERV1, LTR-Gypsy) are disproportionately retained in the somatic genome.

The ~12 Gb of largely repetitive sequence eliminated from somatic cells during chromatin diminution could be either a random or a non-random 80% subset of the germline genome. To discriminate between these two possibilities, we first tested whether some types of TEs were more likely to be excised from the somatic genome than others. Relative frequencies or sample proportions of individual TE superfamilies (i.e. the number of reads mapping to a TE superfamily divided by the total number of reads) are summarized for both genomes in Figure 5. Of the known TE superfamilies, LTR-Gypsy, LINE-L1, and DNA-hAT are present at the highest relative frequencies in both genomes. The ratio of germline to somatic relative frequency ${\hat{f}}_{g}/{\hat{f}}_{s}$ is highly variable across known TE superfamilies, ranging from 0.034 to 4.66 (Table 2; Figure 5). These results suggest that different repeat elements are differentially targeted for excision during chromatin diminution; ${\hat{f}}_{g}/{\hat{f}}_{s}$ >> 1 indicates repeats that are disproportionately excised from the soma, whereas ${\hat{f}}_{g}/{\hat{f}}_{s}$ << 1 indicates repeats that are disproportionately retained in the soma (see Methods). Among the known TE superfamilies, DNA transposon and non-LTR retrotransposon (e.g. LINE) superfamilies that were observed most frequently in the germline genome (${\hat{f}}_{g}$ > 1e-3, i.e. DNA-hAT, DNA-MuDR, LINE-L1) are disproportionately excised from the soma. In contrast, LTR retrotransposons found at higher relative frequencies in the germline genome are disproportionately retained in the soma (LTR-Gypsy) or are retained at near-equal proportions (LTR-Pao). Elements found at lower relative frequencies in the germline genome are almost all disproportionately retained in the soma, although confidence intervals for such elements are large (Figure 5).

**Table 2:** **Results of relative frequency comparisons of repeat superfamilies in the somatic and germline genomes of *M. edax***

**Repeat superfamily**	***H***_**0**_ **:** ***f***_***g***_ **=** ***f***_***s ***_**(P-value)**	**Dominant**	${\hat{f}}_{g}:{\hat{f}}_{s}$ **(Ratio)**	**Threshold *λ *** **(95%)**	**Threshold *λ *** **(99%)**
DNA-Chapaev	0.653	Neither	0.671	NA	NA
DNA-Ginger1	5.11e-5	Somatic	0.0419	< 0.0857	< 0.113
DNA-Harbinger	8.66e-7	Somatic	0.0894	< 0.116	< 0.144
DNA-hAT	<< 1e-16	Germline	1.888	> 1.886	> 1.885
DNA-Kolobok	0.010	Somatic	0.0839	< 0.232	< 0.426
DNA-MuDR	<< 1e-16	Germline	4.66	> 4.56	> 4.48
DNA-Polinton	0.105	Neither	0.484	NA	NA
DNA-Sola	1.04e-5	Somatic	0.194	< 0.233	< 0.269
LINE-CR1	<< 1e-16	Somatic	0.119	< 0.1195	< 0.1200
LINE-L1	<< 1e-16	Germline	2.827	> 2.826	> 2.826
LINE-MINIME_DN	<< 1e-16	Somatic	0.166	< 0.169	< 0.170
LINE-Penelope	0.033	Somatic	0.168	< 0.535	NA
LTR-BEL	0.264	Neither	1.19	NA	NA
LTR-Copia	0.139	Neither	0.707	NA	NA
LTR-DIRS	3.52e-6	Somatic	0.0335	< 0.0639	< 0.0840
LTR-ERV1	<< 1e-16	Somatic	0.147	< 0.148	< 0.149
LTR-ERV3	0.072	Neither	0.252	NA	NA
LTR-Gypsy	<< 1e-16	Somatic	0.5324	< 0.5325	< 0.5325
LTR-Pao	0.042	Germline	1.05	> 1.01	NA

The first data column gives the *P*-value based on a *x*^2^ approximation for rejecting the null hypothesis of equal relative frequencies in the two genomes. The second column identifies which genome has a statistically significant higher relative frequency (α = 0.05) The third column gives the ratio of the observed relative frequencies in the experiment. The fourth and fifth columns give 95% and 99% confidence bounds on the ratio, respectively, for a one-sided test.

**Figure 6:**
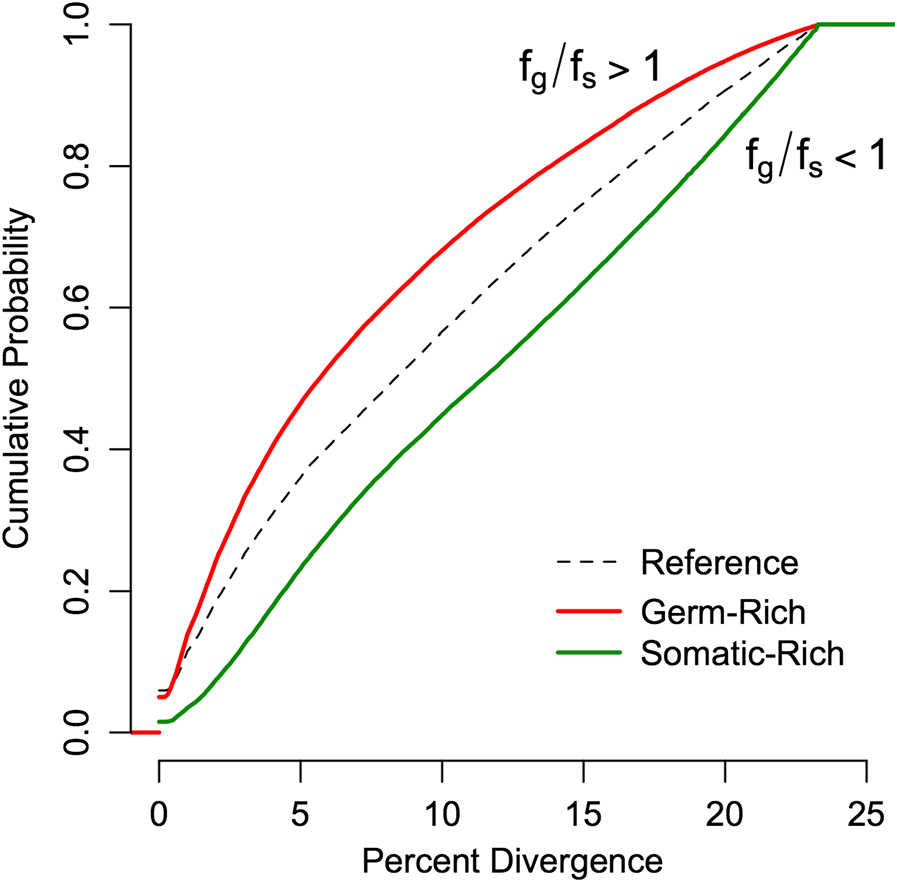
**Cumulative probability distributions of repeat element sequence divergence**/**age.** Reference (dashed line) summarizes sequence divergences of elements present at equal frequencies in germline and somatic genomes (i.e. not disproportionately deleted from, or retained in, the somatic genome during chromatin diminution). Red line shows the distribution of elements present at significantly higher frequencies in the germline than soma; these elements are disproportionately deleted during chromatin diminution. They are significantly younger (less divergent) than the reference elements. Green line shows the distribution of elements present at significantly higher frequencies in the soma than in the germline; these elements are disproportionately retained in the somatic genome during chromatin diminution. They are significantly older (more divergent) than the reference elements. Thus, chromatin diminution disproportionately targets younger repeat elements.

Next, we tested whether repeat elements of certain ages (i.e. sequence divergences) were more likely to be excised from the somatic genome than others. Sequences that are disproportionately eliminated from the somatic genome (${\hat{f}}_{g}/{\hat{f}}_{s}$ >> 1; see Methods) are significantly younger than sequences that are present at equal frequencies in the somatic and germline genomes (${\hat{f}}_{g}/{\hat{f}}_{s}$ = 1) (mean divergence percentages 7.57% and 8.83%, respectively; 2-sample Z-statistic p << 1e-15) (Figure 6). Similarly, sequences that are disproportionately retained in the somatic genome (${\hat{f}}_{g}/{\hat{f}}_{s}$ << 1) are significantly older than sequences that are present at equal frequencies in the somatic and germline genomes (${\hat{f}}_{g}/{\hat{f}}_{s}$ = 1) (mean divergence percentages 10.86% and 8.83%, respectively; 2-sample Z-statistic p <<1e-15) (Figure 6). These results suggest that younger elements are disproportionately deleted from the somatic genome during chromatin diminution, whereas older elements are disproportionately retained.

## Discussion

Our analyses of the first large-scale genomic sequence datasets from copepod germline and soma show that the vast majority of the excised sequences are non-simple, non-tandem (within the length of a single read) repeats of known TE or likely TE origin. We show that 1) young, rapidly proliferating repeat elements underlie the gigantism of adult germline genomes of *M. edax*, and 2) the younger elements are disproportionately excised from the somatic genome. Thus, *M. edax* represents an unusual host/genetic parasite system; some of the deleterious effects of a recent/ongoing explosion in transposition activity (e.g. replication burden) are largely restricted to the germline and substantially reduced in the soma through physical removal of the transposing sequences. Previous smaller scale restriction digestion, cloning, and PCR-based studies of chromatin diminution in copepods identified several short simple repeats, several TEs, rRNAs, and dispersed motifs among the excised DNA sequences [15-17]. However, given the tiny fraction of the genome analyzed in these studies, a comprehensive picture of the targets of chromatin diminution has remained unknown. Our results confirm that all of these types of sequences are eliminated from the somatic genome. However, our conclusions about the relative contributions of these different sequence classes to the excised DNA differ, likely reflecting the different size scales of our datasets. For example, based on analyses of ~200 sequences from pre- and postdiminuted *M. edax* genomes, with read lengths of 500 – 750 bps, McKinnon and Drouin [17] concluded that TEs were not disproportionately deleted during chromatin diminution. The TE sequences in that study were from the LTR/BEL, LTR/Copia, LTR/Gypsy, and DNA/Ginger superfamilies. Our results corroborate their finding that none of these superfamilies is disproportionately excised from the soma; however, other TEs (e.g. DNA-hAT, DNA-MuDR, LINE-L1), as well as unknown repeats, not represented in McKinnon and Drouin’s dataset but represented in our shotgun data are disproportionately excised (Figure 5).

Germline genomic gigantism and chromatin diminution have co-evolved independently several times in the copepod genera *Mesocyclops*, *Metacyclops*, *Megacyclops*, and *Cyclops*[8,26], suggesting that this mechanism of controlling repeat element load has been re-deployed numerous times within the cyclopoid copepods. Based on our current results, we propose the following hypothetical evolutionary scenarios for the origins of this trait: 1) Some copepod lineages begin to experience elevated germline repeat element proliferation, which may reflect genomic invasion of novel elements (note high levels of unknown repeats; Figure 2), decreased efficiency of cellular machinery targeting existing repeats, and/or decreased efficacy of selection against repeats because of strong genetic drift (e.g. a demographic history that includes a population bottleneck). 2) Lineages begin to eliminate these young repeats from the somatic genome by the introduction and non-homologous repair of double-strand breaks at germline-soma differentiation [3]. Because these sequences are eliminated synchronously, an active, enzymatic mechanism is more likely than one mediated by ectopic recombination. Older, more divergent repeat elements (including those that predate the germline genome expansion, as well as those that diverge beyond recognition by excision machinery) remain in the somatic genome (Figure 6). Alternatively, the introduction and non-homologous repair of double-strand breaks at germline-soma differentiation may initially have targeted other sequences not identified in our dataset (e.g. single- or low-copy protein-coding genes), and TEs targeting such regions tagged for excision subsequently proliferated in the germline genome. 3) In either case, the germline and somatic genomes are now free to diverge from one another in repeat element content. In copepods with chromatin diminution, the germline genome grows to 5 – 75 times larger than the somatic genome.

Repeat elements and the repeat suppression/elimination machinery of their hosts co-evolve (e.g. TEs and small RNA-mediated silencing) [27]. The outcome of this coevolutionary dynamic — the amount of repeat sequence present in a genome — is dictated in part by the relative strengths of 1) genetic drift, which can cause fixation of slightly deleterious repeats, and 2) selection for/against repeat insertions and efficacy of the cell’s repeat silencing machinery [28-30]. Our results show that, in *M. edax*, genomes with both high and low repeat content exist within single individuals. Germline genomic gigantism in copepods has been hypothesized to sequester the nucleotides necessary to provision a rapidly differentiating embryo, particularly in nitrogen- and phosphorous-poor environments [9]. This function is a candidate target for selection to increase (or to maintain non-adaptively increased) germline repeat load in *M. edax*, despite the high costs that accompany high TE load (e.g. susceptibility to gain-of-function mutations, deleterious TE insertions, and ectopic recombination; disruption of cellular processes; and potential energetic costs) [31-34]. Alternatively, the high levels of germline repeats may serve no function in *M. edax,* but simply reflect selection’s inability to regulate these “selfish” sequences [35]. In either case, elimination of the majority of repeats through chromatin diminution minimizes TE-associated costs to the soma and maintains ancestral somatic developmental rate, nucleus size, and cell size [20].

Molecular mechanisms of repeat removal in copepods remain unexplored. However, our results in *M. edax*, coupled with information about chromatin diminution in ciliates, suggest a candidate mechanism. In ciliates, the DNA excised from the micronucleus is derived from TEs, and its excision is directed by small-RNA-mediated modification of heterochromatin, followed by excision of this tagged heterochromatin by domesticated transposases [7,13,36,37]. This represents the evolution of a novel function in ciliates for the RNAi-mediated transposon silencing machinery widely shared by eukaryotes [13]. In *M. edax*, the germline genome has extremely high levels of TE and putatively TE-derived sequences (Figure 2), and their sequence divergence distributions show extensive recent and ongoing proliferation (Figure 4). High-frequency DNA transposon and LINE superfamilies, as well as the younger copies of all types of elements, are disproportionately excised from the somatic genome (Figures 5, and 6). Taken together, these results suggest that the RNAi transposon silencing machinery in the ancestral lineages leading to extant taxa with chromatin diminution may have changed function, from transposition silencing (the ancestral condition) to tagging specific TE sequences for excision (the derived condition). Alternatively, repeat removal may occur by a completely different mechanism in *M. edax* and the other cyclopoid copepods that possess chromatin diminution. Further analyses of the molecular processes underlying repeat removal in copepods will demonstrate whether phylogenetically distant taxa have evolved to eliminate repeats by the same, or different, mechanisms.

## Conclusions

Our study used genomic shotgun sequence data from the cyclopoid copepod *Mesocyclops edax* to identify the repetitive sequences present in the species’ massive germline genome, but eliminated from the somatic genome by chromatin diminution. We show that excised repeats are a non-random sample of the total germline genome; younger repeats, as well as high-frequency DNA transposon and LINE superfamilies, are disproportionately excised from the somatic genome. Sequence divergence of repeat elements indicates recent/ongoing repeat proliferation. Taken together, our results suggest that germline genome expansion in *M. edax* reflects explosive proliferation of repeat elements (including known TEs), and that billions of base pairs of such repeats are deleted from the somatic genome every generation. Thus, we hypothesize that chromatin diminution is a mechanism that controls repeat element load, and that this load can evolve to be highly divergent between tissue types within single organisms.

## Methods

### Specimen information, tissue dissection, and genomic DNA extraction

Genomic DNA from somatic and germline cell lineages was obtained from adult female *Mesocyclops edax* Forbes, 1890 collected from Lake Shenandoah, Rockingham Co., Virginia, USA, (38°37’N; 78°83’W). Vouchers of this population are deposited in the National Museum of Natural History (USNM1121766). Somatic cells were obtained from first antennae severed at the 1st or 2nd antennal segment (Figure 1A). Cells containing the undiminuted (i.e. germline) genome were obtained from sacs of 8-cell embryos, which the adult carries external to its body (Figure 1B). Most embryos were first observed at the 4-cell stage and then followed until they had completed more than one half of their development at the 8-cell stage. Samples were preserved in 95% ethanol. Genomic DNA was extracted using the PROMEGA Total RNA isolation kit, omitting the DNAse step, as, in our experience, this kit yields high quality DNA from minute samples. From ~50 females, ~300 and 700 somatic cells and ~2000 and 1000 germ cells were harvested for shotgun sequencing and qPCR analyses, respectively.

### Shotgun library creation and sequencing

Because obtaining genomic DNA from microscopic copepods is very labor intensive, the Repli-G Mini Kit (Qiagen) was used to amplify each genome with minimal bias for coding and satellite DNA [38]. Libraries were prepared using the Nextera DNA Sample Prep Kit (Roche Titanium compatible). Libraries were sequenced on the Roche 454-FLX platform with XLR 70 Titanium reagents. Three-quarters and ¼ of a plate were allocated to germline and soma, respectively, because of genome size differences. Coverage was 0.76% and 1.6% for the germline and somatic genomes, respectively. DNA amplification, library preparation, and sequencing were performed by the University of Idaho Institute for Bioinformatics and Evolutionary Studies (IBEST) Genomics Resources Core facility.

### Initial data processing

Shotgun reads from germline and soma were checked for sequencing artifacts generated by the presence of multiple beads and a single template in emPCR drops, which can skew estimates of repeat element abundance [39,40]. The online 454 Replicate Filter (http://microbiomes.umms.med.umich.edu/replicates/) was used to filter out exact replicate reads (cutoff 0.99, length requirement 1.0 and initial base pair match 3). In total, 2.1% and 2.5% of shotgun reads were removed from the germline and somatic datasets, respectively, as potential artifacts.

### Mining and classification of repeat elements

The pipeline included the following steps: 1) RepeatScout [41] was used to identify *de novo* repeats from shotgun reads, with default parameters. Shotgun reads from germline and soma were combined to increase the sequencing coverage. Identified repeats that were ≤ 50 nt or > 50% low-complexity were removed to construct a filtered RepeatScout library. 2) Shotgun reads were assembled into contigs using Newbler (http://contig.wordpress.com/table-of-contents/) with default parameters. To identify contigs that represent TEs, contig sequences were used as queries to BLASTx against the amino acid sequences of TE-encoded proteins (http://www.repeatmasker.org/RepeatProteinMask.html#database), with an e-value threshold cutoff of 1e-10. Contigs representing TEs were refined manually to avoid assembly artifacts [23]. 3) Repeats identified in step 1 were classified using BLASTn against the TE contigs identified in step 2, with an e-value cutoff of 1e-5. Remaining unclassified repeats were used as queries to tBLASTx against the most recent release of RepBase (RepBase16.12), with an e-value threshold of 1e-5. 4) All classified repeats and TE contigs, along with the unclassified repeats (referred to as “unknown repeats” hereafter), were combined to produce an *M. edax*-specific repeat library. Using this library, we masked the shotgun reads from both germline and soma with RepeatMasker (http://www.repeatmasker.org/). Simple repeats were identified using the Tandem Repeats Finder [42] module in RepeatMasker.

### Experimental verification of repeat content difference between germline and soma

For each of four types of repeats, we picked one representative family that our bioinformatic analyses identified as differing in copy number between germline and soma — DNA/hAT (DNA transposon), LINE/L1 (non-LTR retrotransposon), LTR/Gypsy (LTR retrotransposon), and an ORF-containing unknown repeat. For each family, qPCR primers were designed based on the RepeatScout-generated consensus sequence using Primer3Plus (http://primer3plus.com/cgi-bin/dev/primer3plus.cgi). We amplified a ~200 bp diagnostic fragment from each repeat family using standard PCR from somatic genomic DNA collected and extracted in 2012 using custom primers (Additional file 4). These fragments were cloned into the PCR^®^4-TOPO^®^ vector using a TOPO^®^ TA Cloning^®^ Kit (Invitrogen Life Science Technologies). Plasmids that contained an insert were linearized by digestion with *Spe*I (New England Biolabs), gel-purified, and quantified with a NanoDrop spectrophotometer (Thermo Scientific). Plasmid number per unit volume was estimated based on the molecular weight of plasmid plus insert. A 10-fold dilution series (500,000 to 50 plasmids/μl) of the cloned fragments was used to generate standard curves. Germline and somatic sample DNA was diluted to 50 genomes/ul based on the estimated genome size (germline, 15 Gb; soma, 3 Gb). Quantitative PCR assays were performed in duplicate using 1 ul of sample DNA or cloned DNA (for standard curves) with a LightCycler^®^ 480 Real-Time PCR System following manufacturer’s protocols (Roche Applied Science).

### Characterization of unknown repeat sequences

The longest potential ORFs were identified using two sets of parameters: relaxed and strict. For the relaxed analysis, an ORF was defined as a sequence >100 nucleotides in length that begins with a start codon (ATG) and does not have an in-frame stop codon (TAA, TAG or TGA). For the strict analysis, an ORF was defined as a sequence >100 nucleotides in length that begins with a start codon and ends with an in-frame stop codon. Custom Perl scripts were used to implement these searches. To test whether any unknown repeats are derived from known protein-coding genes, unknown repeats were used as queries to BLASTx against the protein database for *Daphnia pulex* (the closest relative of *M. edax* with a fully sequenced and well-annotated genome) [43], with an e-value threshold of 1e-5. BLASTx against the manually curated UniProt database was also performed, although this search returned slightly fewer hits; thus, only *Daphnia* results are presented.

### Repeat element sequence divergence in somatic and germline genomes

To summarize global historical patterns of repeat proliferation and deletion in *M. edax*, we estimated divergences of repeats from their ancestral sequences (i.e. ancestor–descendant pairwise divergences) in the somatic and germline genomes. For each repeat element family, we first used RepeatScout [41] to estimate a consensus sequence from all genomic repeat copies; this consensus represents the repeat family’s master gene (i.e. ancestral) sequence. Next, we used this set of consensus sequences to mask the germline and somatic datasets with RepeatMasker (http://www.repeatmasker.org/), generating ancestor–descendant pairwise alignment files. We note that some of the sequences identified as confamilial may have been generated by multiple active master genes that differed from one another in sequence. In such a case, a single consensus sequence would not accurately represent the ancestral state of all individual element copies; some of the differences between “ancestor” and descendant sequences would correspond to substitutions that occurred along the active master element lineage. This would produce upwardly biased estimates of sequence divergence. To minimize this problem, we parsed the RepeatMasker-generated pairwise alignments to identify substitutions that likely occurred along active master element lineages, and we excluded these sites from our estimates of sequence divergence. This was done using the following steps. First, we collected all of the pairwise alignments between a given consensus sequence and its descendants in both germline and somatic genomes. To increase accuracy, we limited our analyses to repeat elements ≥ 80% identical to their respective consensus sequences, with a minimum overlap of 100 bp. Second, based on each pairwise alignment, we recorded information about substitutions (i.e. ancestral and derived base pair, position). Third, we compared such substitution information across all pairwise alignments of a given consensus sequence to identify substitutions shared among multiple repeat copies. The probability of two identical substitutions occurring independently in different repeat copies is low; thus, groups of two or more substitutions shared by two or more repeat copies likely reflect substitutions that occurred along the ancestral master element lineage. We did not consider substitutions occurring at CpG sites because of a high probability of convergence at such rapidly evolving sites. Fourth, we updated the pairwise divergence estimates from RepeatMasker, excluding the sites corresponding to shared substitutions. From these refined pairwise alignments, we estimated sequence divergence, correcting with the Jukes-Cantor model of nucleotide substitution. This process was automated using in-house Perl scripts, which are available upon request. We plotted the fraction of total shotgun reads as a function of sequence divergence from consensus; assuming equal rates of nucleotide substitution, such sequence divergence distributions are a proxy for age distributions. We tested for a difference between the sequence divergence cumulative probability distributions of germline and soma using a Kolmogorov-Smirnov test.

To verify that ~1% shotgun coverage gives sufficiently accurate estimates of sequence divergence distributions to support the main conclusions of this study (i.e. high proportions of elements minimally diverged from consensus), we randomly extracted 1% of the human genome five times in read lengths comparable to our 454 data using in-house C code (available upon request), calculated repeat element sequence divergence distributions as for the *M. edax* data, and compared them to published results for the fully sequenced genome.

### Frequency estimates of repeat elements

The frequency of each repeat was analyzed within both genomes in a likelihood framework, which provides a unifying approach to both confidence interval estimation and hypothesis testing [44]. We assumed that each sequence represented an independent Bernoulli trial with success parameter *p* = *f*, where *f* is the underlying probability for the repeat to be observed in a single sequence read from the genome; this assumes that shotgun sequences are an unbiased representation of the genome. When considering the somatic and germline genome datasets separately, with *x*_*s*_ and *x*_*g*_ observed repeats out of *N*_*s*_ and *N*_*g*_ total sequences sampled, the likelihood of the underlying probabilities *f*_*s*_ and *f*_*g*_ is computed as

$$L\left(f_{s},f_{g};x_{s},x_{g}\right)=f_{s}^{x_{s}}{\left(1-f_{s}\right)}^{N_{s}-x_{s}}f_{g}^{x_{g}}{\left(1-f_{g}\right)}^{N_{g}-x_{g}}$$

The maximum likelihood (ML) estimates are the observed relative frequencies, or sample proportions, ${\hat{f}}_{s}=\frac{x_{s}}{N_{s}}$ and ${\hat{f}}_{g}=\frac{x_{g}}{N_{g}}$. The null hypothesis *H*_0_: *f*_*s*_ = *f*_*g*_ leads to a pooled ML estimate ${\hat{f}}_{0}=\frac{x_{s}+x_{g}}{N_{s}+N_{g}}$.

### Characterization of sequences excised during chromatin diminution

Given two pairs of parameter estimates $\left({\hat{f}}_{s,0,}{\hat{f}}_{g,0}\right)$ and $\left({\hat{f}}_{s,1,}{\hat{f}}_{g,1}\right)$, we define the likelihood ratio, $\Delta \left({\hat{f}}_{s,0,}{\hat{f}}_{g,0};{\hat{f}}_{s,1,}{\hat{f}}_{g,1}|x_{s,}x_{g}\right)=\frac{L\left({\hat{f}}_{s,0,}{\hat{f}}_{g,0};x_{s,}x_{g}\right)}{L\left({\hat{f}}_{s,1,}{\hat{f}}_{g,1};x_{s,}x_{g}\right)}$. Joint confidence regions for *f*_*s*_ and *f*_*g*_ can be constructed from likelihood ratios by finding a suitable *κ*^2^ and requiring that $\begin{array}{l} -2\mathrm{ln\Lambda}\left({\hat{f}}_{0,}{\hat{f}}_{0};{\hat{f}}_{s,}{\hat{f}}_{g}|x_{s},x_{g}\right)\geq \kappa^{2} \end{array}$[44]. The cut-off *κ*^2^ was determined using simulations of binomial random variables to represent repeat frequencies with success probabilities based on the observed relative frequencies ${\hat{f}}_{s}$ and ${\hat{f}}_{g}$. For each simulation, the likelihood ratio was computed and the cutoff *κ*^2^ was chosen so that 5% of the simulated values exceeded the cut-off. For relative frequencies greater than 1e-4, the cut-off computed by simulation agreed with cut-offs computed using the usual *χ*^2^ -distribution with 2 degrees of freedom based on a normal approximation. Hypothesis tests with a rejection criterion *X*^2^ ≥ *κ*^2^ were also performed where the test statistic based on the likelihood ratio $\begin{array}{l} X^{2}=-2\mathrm{ln\Lambda}\left({\hat{f}}_{s,0},{\hat{f}}_{g,0};{\hat{f}}_{s,1},{\hat{f}}_{g,1}\;|x_{s},x_{g}\right) \end{array}$ where $\begin{array}{l} \left({\hat{f}}_{s,0},{\hat{f}}_{g,0}\right) \end{array}$ are ML estimates assuming *H*_0_ and $\begin{array}{l} \left({\hat{f}}_{s,1},{\hat{f}}_{g,1}\right) \end{array}$ are ML estimates assuming *H*_1_. Again, the threshold *κ*^2^ was chosen from simulations using the observed relative frequencies to generate binomial random variables in order to obtain the desired probability of a Type 1 error.

We computed 95% joint-confidence regions for *f*_*s*_ and *f*_*g*_. Because these regions are two-dimensional, we created one-dimensional summaries and depicted them in the form of confidence intervals. When these intervals do not overlap, the joint confidence region clearly excludes *f*_*g*_ = *f*_*s*_. However, even when the intervals do overlap, the actual confidence region may be such that *f*_*g*_ = *f*_*s*_ would still be excluded. Thus, we performed hypothesis tests using the likelihood ratio for each superfamily with a null hypothesis *H*_0_: *f*_*g*_ = *f*_*s*_, which is equivalent to $\frac{f_{g}}{f_{s}}=1$, and an alternative hypothesis *H*_1_: *f*_*g*_ ≠ *f*_*s*_, which is equivalent to $\frac{f_{g}}{f_{s}}\neq 1$ with a cutoff *κ*^2^ chosen for a Type 1 error with probability *α* = 0.05. In addition, *P*-values for the test statistic *χ*^2^ were computed based on the percentile ranking of *χ*^2^ relative to simulated data.

For each superfamily that rejected the null hypothesis, we further constructed a one-sided confidence interval for the ratio *f*_*g*_/*f*_*s*_. For a superfamily with $\begin{array}{l} {\hat{f}}_{g}<{\hat{f}}_{s} \end{array}$, we considered the family of null hypotheses $H_{0}\left(\lambda \right):\frac{f_{g}}{f_{s}}\geq \lambda$ and alternative hypotheses $H_{1}\left(\lambda \right):\frac{f_{g}}{f_{s}}<\lambda$ for *λ* < 1 and identified the smallest value *λ* such that the null hypothesis is rejected with *α* = 0.05. For a superfamily with $\begin{array}{l} {\hat{f}}_{g}>{\hat{f}}_{s} \end{array}$, the analogous tests were performed with opposite inequalities and *λ* > 1. The resulting critical relative frequencies correspond to the end-point of a one-sided 95% confidence interval for the ratio $\frac{f_{g}}{f_{s}}$. To demonstrate the sensitivity of this end-point, the process was repeated with α = 0.01, corresponding to producing one-sided 99% confidence intervals for the ratio.

For each repeat subfamily, (including classified and unknown repeats), we first computed the sample relative frequency within each genome (# subfamily reads / total reads). We then computed the ratio of relative frequencies between germline and soma $\begin{array}{l} {\hat{(f}}_{g}/{\hat{f}}_{s})\; \end{array}$ as a measure of relative abundance. This measure yields information about excision during chromatin diminution; $\begin{array}{l} {\hat{f}}_{g}/{\hat{f}}_{s} \end{array}$ >> 1 indicates repeats that are disproportionately excised from the soma, whereas $\begin{array}{l} {\hat{f}}_{g}/{\hat{f}}_{s} \end{array}$ << 1 indicates repeats that are disproportionately retained in the soma. Repeat subfamilies were binned as 1) failing to reject the null hypothesis $\begin{array}{l} {\hat{f}}_{g}/{\hat{f}}_{s} \end{array}$ = 1, 2) favoring the alternative hypothesis $\begin{array}{l} {\hat{f}}_{g}/{\hat{f}}_{s} \end{array}$ >> 1 (i.e. disproportionately excised from the somatic genome), or 3) favoring the alternative hypothesis $\begin{array}{l} {\hat{f}}_{g}/{\hat{f}}_{s} \end{array}$ << 1 (i.e. disproportionately retained in the somatic genome). Hypothesis tests were likelihood ratio tests performed using a Poisson approximation to the binomial distribution (χ^2^ with 1 df) with a p-value cutoff of p = 0.05. For each bin, we plotted the cumulative probability of element age (percent divergence from consensus) and tested for significant differences between the mean divergence percentages for 1) $\begin{array}{l} {\hat{f}}_{g}/{\hat{f}}_{s} \end{array}$ = 1 and $\begin{array}{l} {\hat{f}}_{g}/{\hat{f}}_{s} \end{array}$ >> 1, and 2) $\begin{array}{l} {\hat{f}}_{g}/{\hat{f}}_{s} \end{array}$ = 1 and $\begin{array}{l} {\hat{f}}_{g}/{\hat{f}}_{s} \end{array}$ << 1 using 2-sample z-statistics.

## Competing interests

The authors declare that they have no competing interests.

## Authors’ contributions

CS, GAW, DBW, HW, and RLM planned the work and cowrote the manuscript. CS and DBW performed the data analysis. CS performed the qPCR. GAW obtained genetic material. HW facilitated sequence data collection. All authors read and approved the final manuscript.

## Supplementary Material

Additional file 1 Numbers of unknown sequences from the combined germline and somatic dataset containing open reading frames of different lengths. Click here for file

Additional file 2 ***Daphnia *** **proteins that received BLASTx hits when unknown *M. edax *** **repeats were used as queries, as well as the number of hits in both genomes.** We emphasize that this is not a comprehensive list of genes identified in our shotgun data. Rather, this is a list of genes that exist in multiple copies in the germline or somatic genome, as identified by our repeat-finding pipeline. Click here for file

Additional file 3 **Pairwise divergence of all repeats in 5 subsamples of the human genome, each of which includes ~1% of the genome in read lengths comparable to the *M. edax *** **dataset.** The distributions show a burst of repeat element activity in the past, consistent with published results from the full genome sequence. Click here for file

Additional file 4 **Primers used for qPCR analysis of repeat elements.** Each primer pair targets a single repeat family within the indicated superfamily. Click here for file
