# Supplementary figures and images for: Billions of basepairs of recently expanded, repetitive sequences are eliminated from the somatic genome during copepod development
Source: BMC Genomics. 2014 Mar 11;15:186. doi: 10.1186/1471-2164-15-186 (PMC4029161; doi:10.1186/1471-2164-15-186)

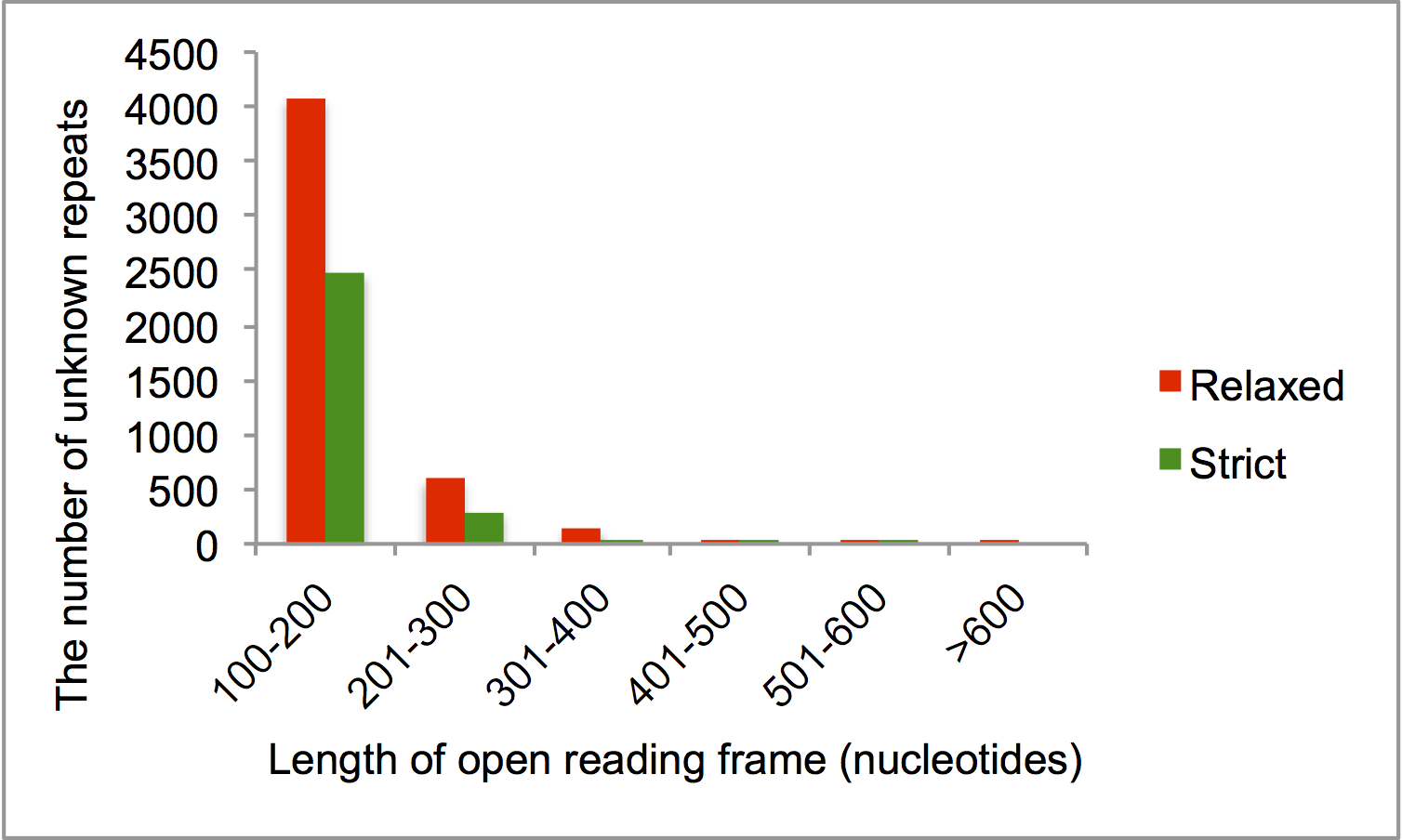

Supplement: Additional file 1 — Numbers of unknown sequences from the combined germline and somatic dataset containing open reading frames of different lengths. [file 1471-2164-15-186-S1.TIFF]

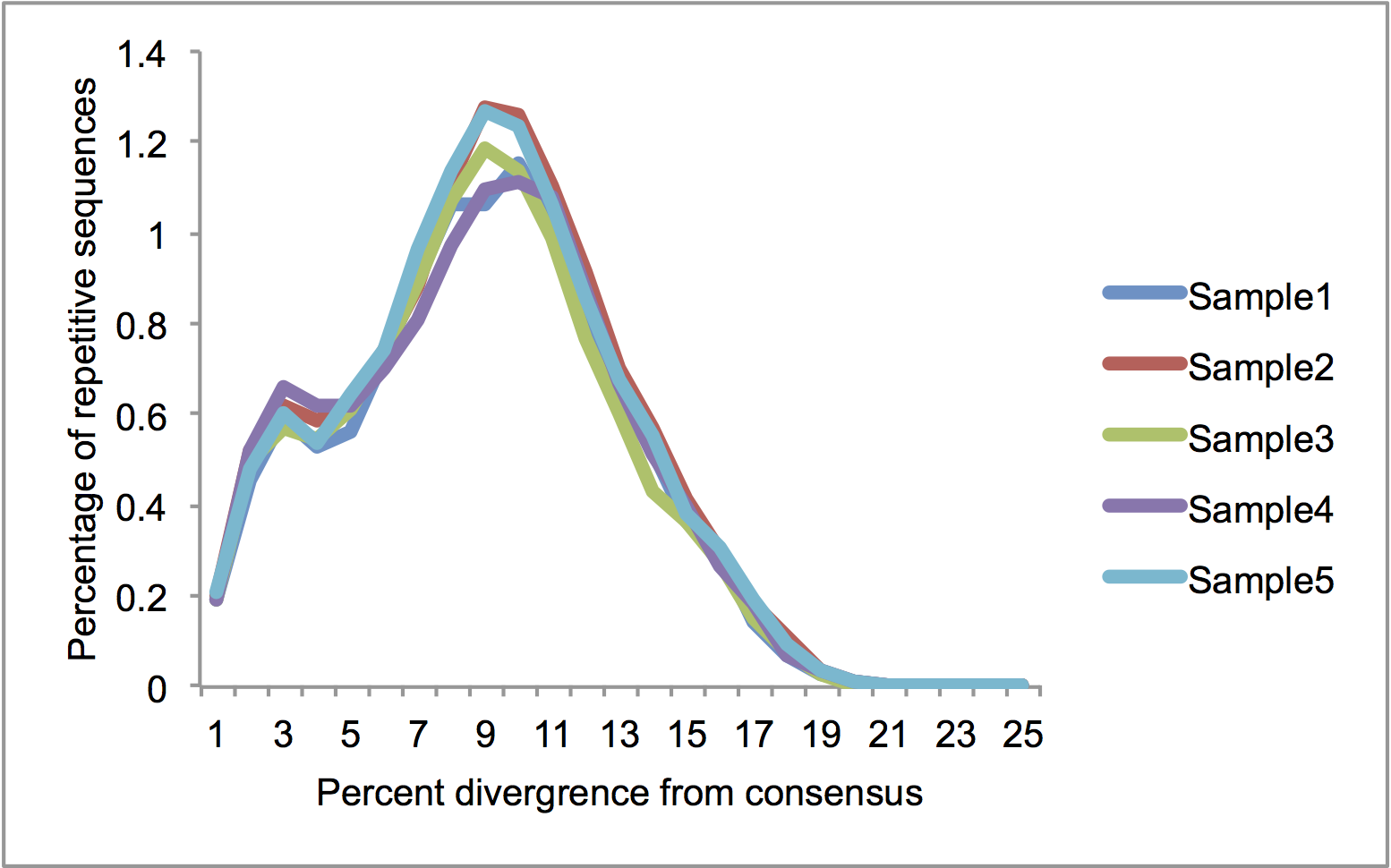

Supplement: Additional file 3 — Pairwise divergence of all repeats in 5 subsamples of the human genome, each of which includes ~1% of the genome in read lengths comparable to the M. edax dataset. The distributions show a burst of repeat element activity in the past, consistent with published results from the full genome sequence. [file 1471-2164-15-186-S3.TIFF]
